# Modulating dynamic brain networks and episodic memory in amnestic mild cognitive impairment using 40 Hz precuneus transcranial alternating current stimulation

**DOI:** 10.1002/alz.092338

**Published:** 2025-01-03

**Authors:** Annel P. Koomen, Cornelis J. Stam, Yolande A.L. Pijnenburg, Willem de Haan

**Affiliations:** ^1^ Amsterdam Neuroscience, Neurodegeneration, Amsterdam, Noord‐Holland Netherlands; ^2^ Alzheimer Center, Amsterdam University Medical Center, location VUmc, Amsterdam, Noord‐Holland Netherlands; ^3^ Clinical neurophysiology and MEG Center, Amsterdam University Medical Center, location VUmc, Amsterdam, Noord‐Holland Netherlands

## Abstract

**Background:**

Worldwide, 32 million Alzheimer’s disease (AD) patients contribute to a large economic burden, making effective and safe therapies that slow or prevent the progression from pre‐dementia or mild cognitive impairment (MCI) to AD of high priority. Transcranial alternating current stimulation (tACS) is a safe and patient‐friendly non‐invasive brain stimulation technique that serves as a potential candidate for slowing and/or reducing cognitive impairment. Application of tACS in the gamma (30‐45 Hz) frequency range, specifically around 40 Hz, has been studied in patients with (pre‐dementia) AD. In these patients, a single session of 40 Hz tACS at the precuneus region showed to improve episodic memory and to increase relative power of higher frequencies such as gamma, as measured with EEG. These findings will be replicated in the current study in pre‐dementia AD patients, using magnetoencephalography (MEG) to study brain network dynamic changes with greater temporal and spatial detail.

**Method:**

In this double‐blind, randomized, sham‐controlled, cross‐over trial, 30 pre‐dementia AD (biomarker‐confirmed amnestic MCI) patients will undergo concurrent tACS‐MEG. At two sessions separated by a week, 48 minutes of either gamma (40 Hz) or sham tACS will be applied at the precuneus region. Cognitive outcome measures assessed pre and post‐tACS are the Rey Auditory Verbal Learning (RAVL) test and Face‐Name Association Task (FNAT) score. In addition, resting‐state spectral and functional connectivity MEG measures will be extracted from the pre‐ and post‐tACS data to determine tACS‐induced network effects.

**Result:**

An increase in RAVL test and FNAT score after 40 Hz but not sham tACS is expected, as well as an increase of relative power and global connectivity in higher frequencies (alpha [8‐13 Hz], beta [13‐30 Hz] and gamma [30‐45 Hz]). Furthermore, a positive correlation is expected between these quantitative MEG measures and cognitive outcome following 40 Hz tACS.

**Conclusion:**

We expect 40 Hz precuneus tACS to improve episodic memory and to counteract the slowing of oscillatory activity and reduction of brain communication in AD. This will support the notion that this approach can temporarily improve cognitive impairment by normalizing disturbed brain function, and encourages the use of tACS in clinical studies of AD.

